# Ethnicity-Based Variations in Focal Adhesion Kinase Signaling in Glioblastoma Gene Expression: A Study of the Puerto Rican Hispanic Population

**DOI:** 10.3390/ijms25094947

**Published:** 2024-05-01

**Authors:** Tyrel Porter, Miguel Mayol del Valle, Lilia Kucheryavykh

**Affiliations:** 1 Department of Biochemistry, Universidad Central del Caribe, Bayamón, PR 00956, USA; lilia.kucheryavykh@uccaribe.edu; 2 Department of Surgery, Neurosurgery Section, University of Puerto Rico, Medical Sciences Campus, San Juan, PR 00921, USA

**Keywords:** glioblastoma, ethnicity, gene expression

## Abstract

Glioblastoma (GBM), an aggressive form of brain cancer, has a higher incidence in non-Hispanics when compared to the US Hispanic population. Using data from RT-PCR analysis of 21 GBM tissue from Hispanic patients in Puerto Rico, we identified significant correlations in the gene expression of focal adhesion kinase and proline-rich tyrosine kinase (PTK2 and PTK2B) with NGFR (nerve growth factor receptor), PDGFRB (platelet-derived growth factor receptor B), EGFR (epithelial growth factor receptor), and CXCR1 (C-X-C motif chemokine receptor 1). This study further explores these correlations found in gene expression while accounting for sex and ethnicity. Statistically significant (*p* < 0.05) correlations with an r value > ±0.7 were subsequently contrasted with mRNA expression data acquired from cBioPortal for 323 GBM specimens. Significant correlations in Puerto Rican male patients were found between PTK2 and PTK2B, NGFR, PDGFRB, EGFR, and CXCR1, which did not arise in non-Hispanic male patient data. The data for Puerto Rican female patients showed correlations in PTK2 with PTK2B, NGFR, PDGFRB, and EGFR, all of which did not appear in the data for non-Hispanic female patients. The data acquired from cBioPortal for non-Puerto Rican Hispanic patients supported the correlations found in the Puerto Rican population for both sexes. Our findings reveal distinct correlations in gene expression patterns, particularly involving PTK2, PTK2B, NGFR, PDGFRB, and EGFR among Puerto Rican Hispanic patients when compared to non-Hispanic counterparts.

## 1. Introduction

Glioblastoma (GBM) is a highly aggressive brain tumor with poor prognosis [1], necessitating a deeper understanding of its molecular mechanisms. Ethnicity and sex have been identified as influential factors in cancer incidence and treatment response [2,3,4]. Studies have reported variations in GBM incidence and survival rates among different ethnic and racial groups, with non-Hispanic populations exhibiting higher incidence rates compared to Hispanic populations [3]. Additionally, epidemiological data yields a 60% higher chance to develop GBM in male patients than in females [5]. The molecular basis for these disparities remains poorly understood. While gene expression changes may not always impact biological activity, identifying differential expression present in pathologies often yields valuable insights into understanding pathological disparities [6]. The need exists for further investigation into ethnicity and sex-based variations in gene expression patterns in GBM.

Focal adhesion kinase (FAK) and proline-rich tyrosine kinase (Pyk2), encoded by PTK2 and PTK2B genes, respectively, have been shown to integrate signals from cell adhesion, growth factors, and G-protein-coupled receptors [7,8]. For this reason, the activation of Pyk2/FAK signaling has been associated with the migration and proliferation of GBM [7,9]. Furthermore, gene expression and protein phosphorylation levels of both Pyk2 and FAK have demonstrated positive correlations in GBM with growth factor receptor expression, including epidermal growth factor receptor (EGFR), platelet-derived growth factor receptor b (PDGFRB), and nerve growth factor receptor (NGFR) [7,10]. The autocrine and paracrine activation of growth factors receptors in glioma cells enhances their migratory capacity, emphasizing the role of PDGFRB, EGFR and NGFR in tumor progression, and has been identified as an additional factor contributing to GBM tumor dispersal, growth, and treatment resistance [8,9,10,11,12,13]. Differentiated expression in growth factor receptors and their correlation with Pyk2/FAK gene expression in GBM may yield information pertinent to the disparities in incidence and treatment response when accounting for ethnicity and sex.

In this study, we investigated ethnicity and sex-based variations in gene expression patterns, focusing on correlations between the genes PTK2, PTK2B, PDGFRB, NGFR, and CXCR1, along with a particular emphasis on EGFR, given its strong association with brain tumors, with the purpose being to elucidate the molecular underpinnings of GBM pathogenesis and address the disparities observed in different patient populations [14].

## 2. Results

**Figure 1:**
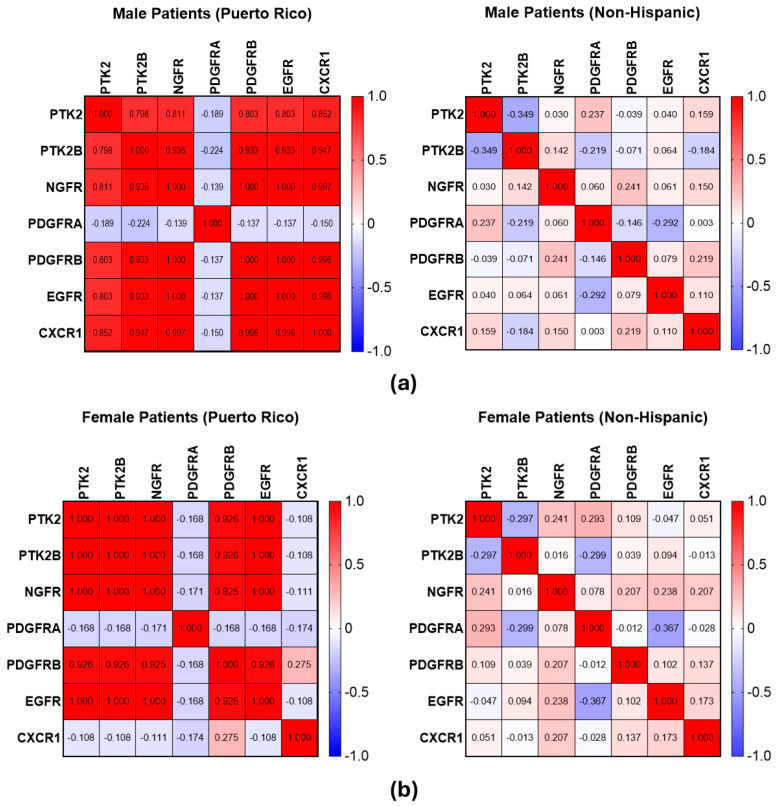
Heat map of Pearson correlation matrix for PDGFRB, NGFR, EGFR, and CXCR1 cell-surface receptors’ gene expression, assessed through RT-PCR, as well as Pyk2 and FAK gene expression in human GBM cells, purified from tumors obtained from a cohort of patients from Puerto Rico (N= 21). The Puerto Rican cohort was contrasted with non-Hispanic patient data from a microarray dataset, containing 323 samples, downloaded from cBioPortal, Firehose Legacy RNA-Seq dataset. (**a**) Pearson correlation matrices comparing Puerto Rican males and non-Hispanic males. (**b**) Pearson correlation matrices comparing Puerto Rican females and non-Hispanic females.

RT-PCR analysis of gene expression data revealed statistically significant (*p* < 0.05) positive correlations among Puerto Rican Hispanic male patients (Figure 1a) between PTK and NGFR (r = 0.811), PTK and PDGFRB (r = 0.848), PTK and EGFR (r = 0.803), and PTK and CXCR1 (r = 0.852). Other correlations arose between PTK2B and PTK, NGFR, PDGFRB, EGFR, and CXCR1 (r = 0.798, 0.935, 0.938, 1.000, and 0.997, respectively). Lastly, NGFR correlated with PDGFRB, EGFR, and CXCR1 (r = 0.998, 1.000, and 0.997, respectively). These same correlations were not expressed in the data from non-Hispanic male patients, obtained from the Firehose Legacy RNA-Seq dataset, cBioPortal (Figure 1b), with statistically significant r values arising between PTK2 and PTK2B (r = −0.349), PDGFRA (r = 0.237), and CXCR1 (r = 0.159), as well as between PTK2B and NGFR (r = 0.142), PDGFRA (r = −0.219), and CXCR1 (r = −0.184). Other statistically significant r values were found between NGFR and PDGFRB (r = 0.241) and CXCR1 (r = 0.150).

Significant positive correlations were discovered in Puerto Rican Hispanic female patients between PTK2 and PTK2B (r = 1.000), NGFR (r = 1.000), PDGFRB (r = 0.926), and EGFR (r = 1.000). Additionally, PTK2B correlated with NGFR, PDGFRB, and EGFR (r = 1.000, 0.926, and 1.000, respectively). NGFR was found to correlate with PDGFRB (r = 0.925) and EGFR (r = 1.000). =As with non-Hispanic male patients, these strong correlations were not observed in non-Hispanic female patients, with statistically significant r values observed between PTK and PTK2B (r = −0.297), NGFR (r = 0.241), and PDGFRA (r = 0.293), as well as between PTK2B and PDGFRA (r = −0.299). Lastly, NGFR had statistically significant correlations with PDGFRB, EGFR, and CXCR1 (r = 0.207, 0.238, and 0.207, respectively).

**Figure 2:**
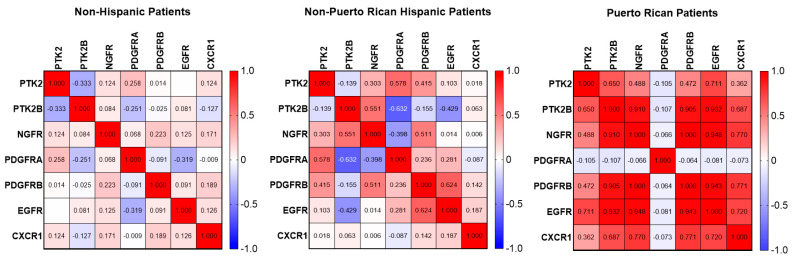
Heat map generated to visualize the Pearson correlation matrix for the gene expression of PDGFRA, PDGFRB, NGFR, EGFR, and CXCR1 cell-surface receptors, as well as Pyk2 and FAK, in human GBM cells. The analysis compares combined data from males and females across non-Puerto Rican Hispanic and non-Hispanic cohorts, with sample sizes of N = 10 and N = 313, respectively, obtained from the cBioPortal Firehose Legacy microarray dataset. Additional data from Puerto Rican patients (N = 21) were obtained through RT-PCR analysis of GBM specimens.

Within the mRNA expression data from 483 GBM specimens sourced from cBioPortal, only 10 patients were of Hispanic ethnicity. These 10 patients were labeled as non-Puerto Rican Hispanic patients. However, given the smaller sample size, patient data were combined for male and female Hispanic patients and acknowledged as a limitation (Figure 2). One statistically significant correlation appeared in the data between PTK2B and PDGFRA (r = −0.632). Some minor positive trends, consisting of r value increases of 0.1–0.4, were observed in the correlation heatmap exhibiting consistencies shown in Puerto Rican Hispanic data. Specifically, increased correlations between PTK2 and NGFR, PDGFRB, and EGFR; between PTK2B and NGFR and CXCR1; and between NGFR and PDGFRB were observed in both male and female Puerto Rican Hispanic Patients when contrasted against a combined correlation heatmap of both non-Hispanic male and female patients.

## 3. Discussion

Our observations align with previous reports highlighting ethnic and biological sex disparities manifested in GBM [2,3,4]. By exploring ethnicity-based variations in gene expression patterns, we identified significant correlations in gene expression patterns involving PTK, PTK2B, NGFR, PDGFRB, and EGFR among male and female Puerto Rican patients and CXCR1 in male Puerto Rican patients. Notably, these correlations were largely absent in non-Hispanic patients, both in males and females.

The observed differences between Puerto Rican men and women in gene expression patterns raise intriguing questions regarding potential underlying mechanisms—most notably, the absence of strong correlations with CXCR1 in women when compared to men. However, some research has demonstrated higher CXCR1 mRNA expression levels in men than women in other pathologies, which is consistent with our data shown in Puerto Rican patients [15]. While our study did not directly investigate the influence of hormones or the specific sequence variations in the studied genes across different ethnicities, these factors could indeed play a role in modulating gene expression patterns in glioblastoma. Studies have shown that estrogen may have a protective effect in preventing GBM [16,17]. Furthermore, neural androgen receptors appear to modulate gene expression in the brain and are upregulated in the presence of testosterone [18,19]. Further analyses exploring the interplay between hormonal factors, genetic variations, and gene expression profiles across diverse ethnic groups could provide deeper insights into the observed disparities.

The Puerto Rican demographic poses a distinctive medical challenge due to its intricate genetic composition, characterized by a blend of Native American, European, and African ancestries [20]. Substantial pathological distinctions emerge between US Hispanics and Puerto Rican Hispanics, even after adjusting for socioeconomic factors, impacting various physiological systems [21,22,23]. Additionally, EGFR demonstrates varying rates of mutation in cancer when considering race and ethnicity, with evidence for a higher incidence of EGFR mutations in Puerto Rican patients compared with Caucasian patients [24,25]. While our study provides valuable insights into ethnicity-based variations in gene expression patterns in glioblastoma, it is essential to acknowledge certain limitations. The significant differences in the number of samples between the studied subgroups may have influenced the occurrence of statistically significant relationships. Additionally, the small sample size available for non-Puerto Rican Hispanic patients limits the relationships that may be inferred between Puerto Rican Hispanics and non-Puerto Rican Hispanics. Whilst acknowledging the constraints of our study, notably the relatively modest sample size of non-Puerto Rican Hispanic participants, existing research indicates a reduced incidence of GBM among Hispanics of Mexican/Central American descent compared to those of Caribbean descent [4]. It is plausible that genetic and pathological distinctions between these two groups contribute to the observed disparities in our data between Puerto Rican patients and their non-Puerto Rican Hispanic counterparts.

Future studies with larger and more balanced sample sizes across different ethnic and gender subgroups would help to validate and extend our findings. Furthermore, initiatives should prioritize a multi-omic approach and the incorporation of people of Hispanic origin, a factor only recently gaining recognition in cancer research [26,27]. This approach would further validate our findings, which have focused only on the gene expression level, to ascertain their clinical significance.

In conclusion, our study offers evidence of ethnicity-based disparities in gene expression profiles within GBM tumors that are present in the Puerto Rican population. Our analysis revealed distinct correlations between the gene expression of focal adhesion kinases (FAK and Pyk2) and growth factor receptors (NGFR, PDGFRB, and EGFR) in Puerto Rican patients, which were notably absent or significantly attenuated in their non-Hispanic counterparts. Additionally, strong correlations were observed between focal adhesion kinases and CXCR1 in male Puerto Rican patients, but not in female Puerto Rican patients. These strong correlations observed in Puerto Rican patients underscore the importance of tailoring treatment strategies to individual patients’ genetic profiles, potentially leading to more effective and targeted interventions. This elucidation of ethnic-specific gene expression patterns highlights the necessity of incorporating ethnic diversity into future research endeavors aimed at advancing personalized cancer therapy.

## 4. Materials and Methods

### 4.1. Tumor Specimens Acquisition

Tumor specimens from patients diagnosed with grade IV glioblastoma (GBM, WHO classification) were sourced from the University District Hospital Rio Piedras Medical Center and the HIMA San Pablo Hospital in Puerto Rico. Informed consent was obtained from all participants, and the study protocol was approved by the Institutional Review Board (IRB) Human Research Subject Protection Office under protocol #2012-12B. The inclusion criteria encompassed individuals aged ≥21 years with confirmed CNS neoplasia through neuroimaging techniques. Pathological analysis confirmed grade IV GBM. Tissue samples, approximately 1.5–2.0 cm^3^ in volume, were promptly collected after surgical dissection from the resected tumor mass. These samples were then utilized within one hour for further analysis.

### 4.2. GBM Cells Purification from Tumor Specimens

The purification of glioblastoma cells from homogenized tumor tissue was carried out using Percoll density gradient centrifugation. Tumor tissue was transported to the lab in ice-cold PBS and processed for the study within 1 h after tumor resection. Percoll (Sigma-Aldrich, St. Louis, MO, USA, cat. #E0414) was employed to create concentration layers of 30%, 37%, and 70%. The glioma fraction was collected from the top of the Percoll layers and used for further analysis as previously described [7].

### 4.3. Real-Time RT-PCR

Total RNA extraction from purified glioma cells from Puerto Rican patients utilized the RNeasy Plus Mini Kit (Qiagen GmbH, Hilden, Germany, cat. #74134). RNA quality and concentration were determined using the NanoDrop 1000 spectrophotometer (Thermo Scientific, Waltham, MA, USA). Reverse transcription was carried out using the iScript cDNA synthesis kit (Bio-Rad, Hercules, CA, USA, cat. #1708890). RNA samples in concentrations of 3–8 ng/μL and demonstrating a 260/280 ratio in diapason 1.8–2.0 were used for RT-PCR analysis. Real-time RT-PCR analysis utilized SsoAdvanced Universal SYBR Green Supermix (Bio-Rad, cat. #725271) and specific primers for target genes, PTK2 (Pyk2), PTK2B (FAK), PDGFRB, EGFR, CXCR1 and NGFR, with GAPDH serving as an endogenous control for normalization (amplification and melting curves for the listed primers are provided in Figure S1). Amplification was carried out in a Bio-Rad CFX96 Touch real-time PCR detection system (Bio-Rad). The gene expression level was defined as the threshold cycle number (CT). The comparative CT method (relative expression units, 2^(−ΔΔCt)^) was used to calculate mean fold changes in gene expression.

### 4.4. Gene Expression Data

Clinical data and expression values for PTK2, PTK2B, PDGFRB, EGFR, CXCR1, and NGFR (Glioblastoma (GBM), The Cancer Genome Atlas Program (TCGA) Provisional for Cancer Genomes, mRNA Expression z-Scores) were obtained from cBioPortal for cancer genomics (http://www.cbioportal.org (accessed on 21 October 2023)), which contains annotated TCGA data [28,29]. Expression values were normalized through means of RSEM prior to downloading [30]. The Glioblastoma Multiforme (TCGA, Firehose Legacy) RNA-seq dataset, containing 323 samples, was directly downloaded from the cBioPortal on 7 February 2023. Gene expression correlation analysis was conducted separately for males and females, as well as for non-Puerto Rican Hispanic and non-Hispanic cohorts using cBioPortal data. These analyses were subsequently contrasted with RT-PCR data obtained from GBM cells in Puerto Rican Hispanic patients.

### 4.5. Statistical Analysis

Pearson correlation analysis was employed to assess the correlation between gene expressions. A *p*-value of <0.05 for correlations arising from multivariable analysis with an r value > ±0.7 was considered significant. GraphPad Prism 9.1.0 software was used for statistical analysis.

## Data Availability

The data generated in this study are available within the article and the Supplementary Materials.

## Supplementary Materials

The following supporting information can be downloaded at: https://www.mdpi.com/article/10.3390/ijms25094947/s1.
