# Supplementary figures and images for: Ethnicity-Based Variations in Focal Adhesion Kinase Signaling in Glioblastoma Gene Expression: A Study of the Puerto Rican Hispanic Population
Source: Int J Mol Sci. 2024 May 1;25(9):4947. doi: 10.3390/ijms25094947 (PMC11084467; doi:10.3390/ijms25094947)

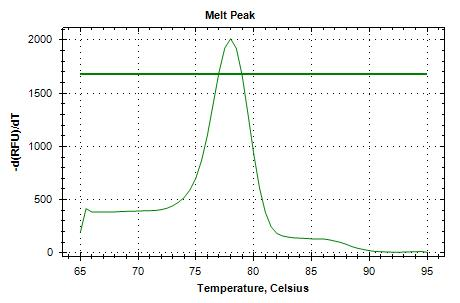

Supplement: Supplementary file 1 [file ijms-25-04947-s001.zip › CXCR1 melting.tiff]

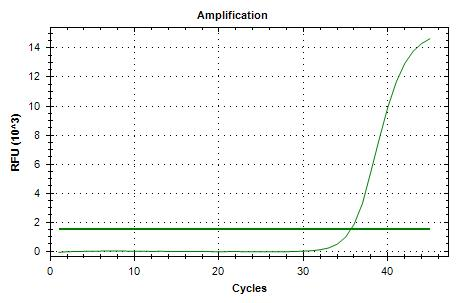

Supplement: Supplementary file 1 [file ijms-25-04947-s001.zip › CXCR1.tiff]

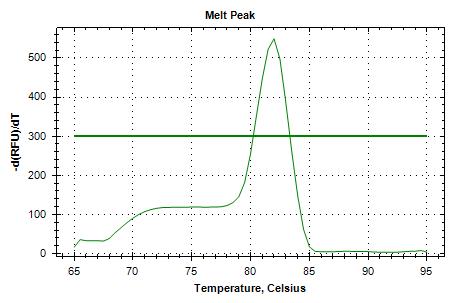

Supplement: Supplementary file 1 [file ijms-25-04947-s001.zip › EGFR melting.jpg]

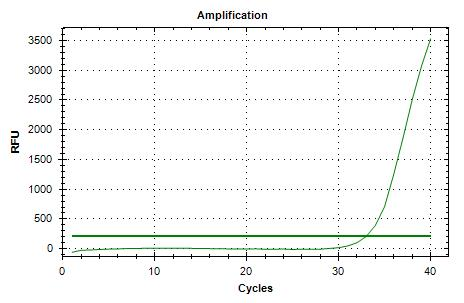

Supplement: Supplementary file 1 [file ijms-25-04947-s001.zip › EGFR.tiff]

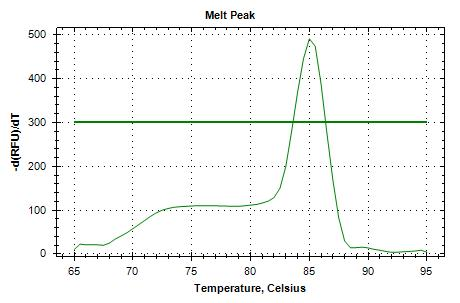

Supplement: Supplementary file 1 [file ijms-25-04947-s001.zip › FAK melting.tiff]

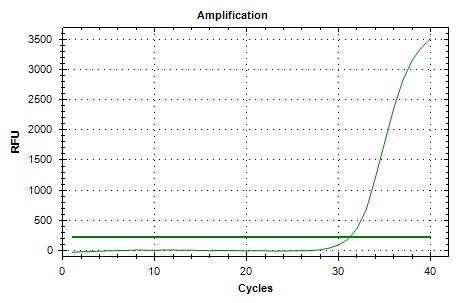

Supplement: Supplementary file 1 [file ijms-25-04947-s001.zip › FAK.tiff]

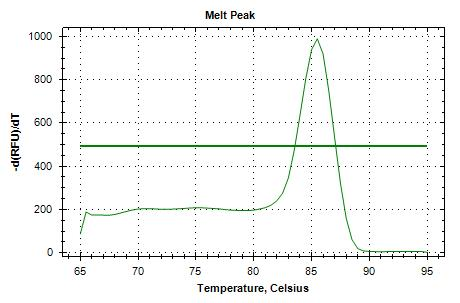

Supplement: Supplementary file 1 [file ijms-25-04947-s001.zip › GAPDH melting.tiff]

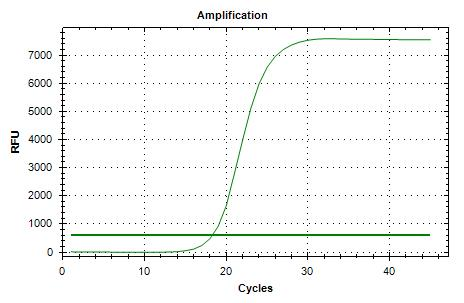

Supplement: Supplementary file 1 [file ijms-25-04947-s001.zip › GAPDH.tiff]

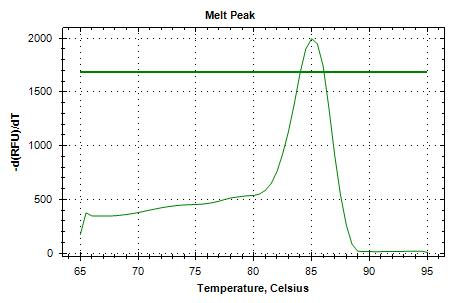

Supplement: Supplementary file 1 [file ijms-25-04947-s001.zip › NGFR melting.tiff]

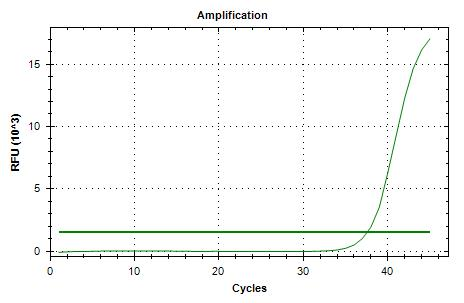

Supplement: Supplementary file 1 [file ijms-25-04947-s001.zip › NGFR.tiff]

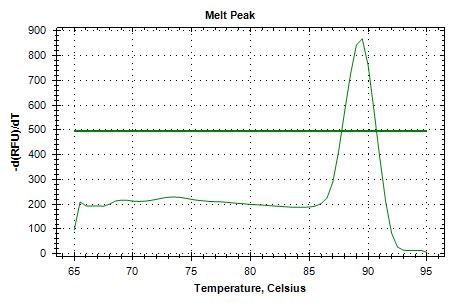

Supplement: Supplementary file 1 [file ijms-25-04947-s001.zip › PDGFRa melting.tiff]

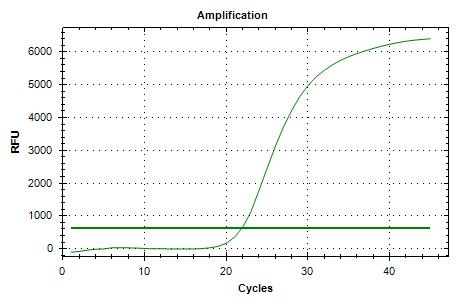

Supplement: Supplementary file 1 [file ijms-25-04947-s001.zip › PDGFRa.tiff]

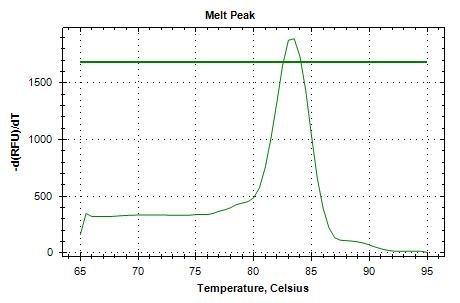

Supplement: Supplementary file 1 [file ijms-25-04947-s001.zip › PDGFRb melting.tiff]

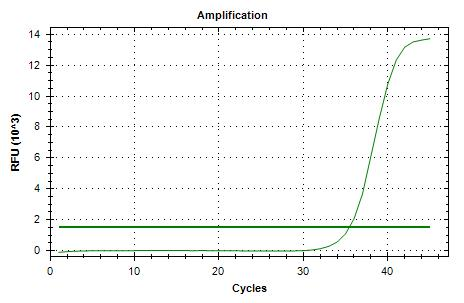

Supplement: Supplementary file 1 [file ijms-25-04947-s001.zip › PDGFRb.tiff]

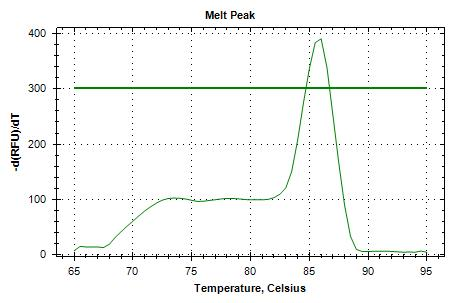

Supplement: Supplementary file 1 [file ijms-25-04947-s001.zip › Pyk2 melting.tiff]

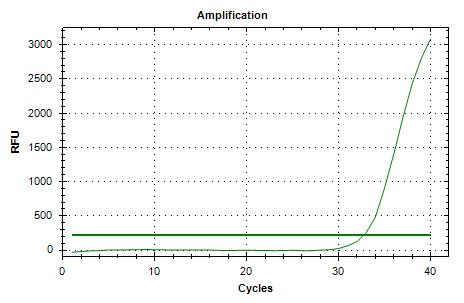

Supplement: Supplementary file 1 [file ijms-25-04947-s001.zip › Pyk2.tiff]
